# An Observational Longitudinal Study on Seasonal Variations in Tourette Syndrome: Evidence for a Role of Ambient Temperature in Tic Exacerbation

**DOI:** 10.3390/biomedicines12081668

**Published:** 2024-07-26

**Authors:** Jacopo Lamanna, Riccardo Mazzoleni, Ramona Farina, Mattia Ferro, Roberta Galentino, Mauro Porta, Antonio Malgaroli

**Affiliations:** 1 Center for Behavioral Neuroscience and Communication (BNC), Vita-Salute San Raffaele University, 20132 Milan, Italy; lamanna.jacopo@hsr.it (J.L.); riccardo_mazzoleni@outlook.com (R.M.); m.ferro@milano-sfu.it (M.F.); 2 Faculty of Psychology, Vita-Salute San Raffaele University, 20132 Milan, Italy; 3 Tourette Center, IRCCS Galeazzi Orthopaedic Institute, 20157 Milan, Italy; ramona.farina@outlook.it (R.F.); roberta.galentino@gmail.com (R.G.); portamilano@libero.it (M.P.); 4 Department of Psychology, Sigmund Freud Private University, 20143 Milan, Italy; 5 Clinical Center Tourette Syndrome, IRCCS Ospedale San Raffaele, 20127 Milan, Italy

**Keywords:** Tourette syndrome, tic disorders, obsessive–compulsive disorder, seasons, temperature, synchrony, Yale global tic severity scale, OCD, TS

## Abstract

Tourette syndrome (TS) is a high-incidence neurobehavioral disorder that generally begins in childhood. Several factors play a role in its etiology, including genetic influence and auto-immune activation by streptococcal infections. In general, symptoms subside after the end of adolescence, but, in a significant number of patients, they remain in adulthood. In this study, we evaluated temporal variations in the two core clinical features of TS including tics and obsessive–compulsive disorder (OCD) symptoms. An observational longitudinal study lasting 15 months (2017–2019) was conducted on a cohort of 24 people recruited in Milan (Italy) who were diagnosed with a subtype of TS known as obsessive–compulsive tic disorder. Inclusion criteria included a global score of the Yale global tic severity scale (Y-GTSS) > 50, a Yale–Brown obsessive–compulsive scale (Y-BOCS) global score > 15, and TS onset at least one year prior. Y-GTSS and Y-BOCS data were acquired at six time points, together with local environmental data. Tics, but not OCD symptoms, were found to be more severe in spring and summer compared with winter and autumn (*p* < 0.001). Changes in tics displayed an appreciable oscillation pattern in the same subject and also a clear synchrony among different subjects, indicating an external orchestrating factor. Ambient temperature showed a significant correlation with Y-GTSS measurements (*p* < 0.001). We argue that the increase in tics observed during hot seasons can be related to increasing ambient temperature. We believe that our results can shed light on the seasonal dynamics of TS symptomatology and provide clues for preventing their worsening over the year.

## 1. Introduction

Seasonal variation in disease incidence and symptomatology is known to characterize several disorders of public health relevance. Several recent studies have confirmed seasonal rhythmicity in symptoms in many different pathologies including infectious diseases, as well as neurological disorders such as headache, multiple sclerosis, ischemic strokes, and Parkinson’s disease (PD) [1,2]. Similarly, circannual oscillations were found to characterize many psychiatric disturbances, including schizophrenia, seasonal affective disorder, panic disorders, generalized anxiety disorder, obsessive–compulsive disorder (OCD) [3], and eating disorders [2]. In the present paper, we evaluated the presence of seasonal oscillations in the frequency and intensity of tics and obsessive–compulsive (OC) symptoms in the subtype of Tourette syndrome (TS) known as obsessive–compulsive tic disorder (OCTD) [4]. This syndrome is characterized by the presence of tics, recurrent and non-rhythmic movements or vocalizations, and obsessive–compulsive symptomatology. TS typically develops before 10 years old, showing a waxing and waning course, usually ameliorating with age. The prevalence is estimated to be almost 1% in children and adolescents. A few studies about seasonality in OCD report an increase in OC symptoms in autumn and winter and a decrease in summer and spring [5]. Regarding tics, until now, a small number of studies have analyzed non-linear trends. In a study by Leckman and colleagues [6], a fractal distribution of tics was observed, i.e., a non-linear relationship of self-similarity across different time scales [7,8]. Despite the importance of this topic, up to now, tic occurrence has mainly been studied by predicting its evolution over the years. In a previous study from our group, conducted on a similar cohort of TS patients, no significant seasonal oscillatory behavior in tics was detected [9]. That investigation was conducted over the course of one year. To further extend that study, we investigated seasonal variations in terms of frequency and intensity of tics and OCD symptoms in patients with OCTD across two years and applied a different analysis approach to the collected data. Using the novel analysis approach, we tried to address the following questions: (i) are there specific seasonal patterns in OC and/or tic symptoms in patients suffering from OCTD? (ii) if present, can these be attributed to temperature and humidity changes? and (iii) are variations in tic and OC symptoms across different subjects correlated or independent?

## 2. Materials and Methods

### 2.1. Subjects

**Figure 1:**
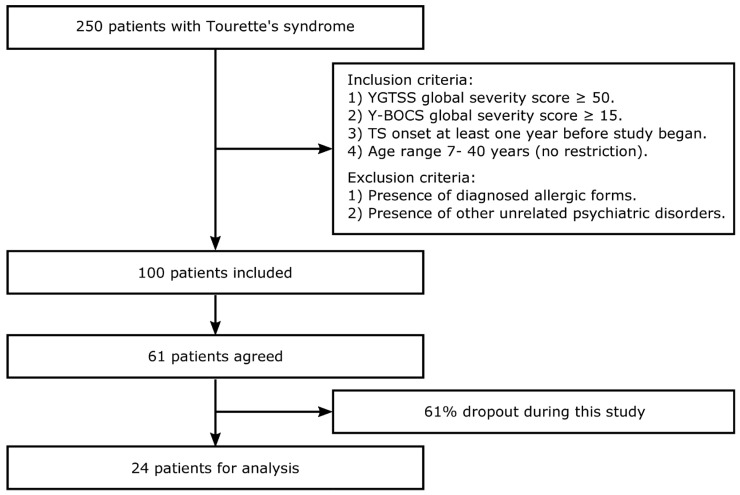
Study flowchart. Criteria and numbers related to patient enrollment, non-inclusions, and drop-outs during this study.

In this study, patients were diagnosed with OCTD by neurological/psychiatric clinical assessments. All patients received different pharmacological treatments during the study period (antipsychotics, antidepressants, and anxiolytics; antibiotic prophylaxis for group A streptococcus based on hematological tests). Yale global tic severity scale (Y-GTSS) [10] and Yale–Brown obsessive–compulsive scale (Y-BOCS) [11] data were acquired by clinical psychologists. Sixty-one participants were recruited (48 males, 13 females; age: 7–40 years (min–max); 18.5 ± 9.15 years (mean ± SD)). The inclusion criteria were (1) the presence of a value equal to or greater than 50 on the global severity score of Y-GTSS at the beginning of the assessment; (2) the presence of a value equal to or greater than 15 on the global severity score of Y-BOCS; and (3) TS onset occurred at least one year before the beginning of this study; (4) no age restrictions were applied (age range 7–40 years). The exclusion criteria were (1) the presence of diagnosed allergic forms and (2) the presence of other psychiatric disorders except those associated with the syndrome. Participants were recruited at the Department of Neurology of IRCCS Galeazzi Hospital (Milan) by MP. In the month of October 2017, each selected subject was contacted, and 61 subjects confirmed their availability to participate in this study. A large percentage of subjects (61%) dropped out during this study. The here analyzed cohort included 24 subjects (male-to-female ratio = 20:4; age mean ± SD = 20.58 ± 9.18 years). Figure 1 shows a flowchart of the patient recruitment and selection process. Patients were tested for Y-GTSS and Y-BOCS in November 2017 (T0), February 2018 (T1), May 2018 (T2), August 2018 (T3), November 2018 (T4), and February 2019 (T5).

### 2.2. Ethical Statement

The San Raffaele Ethics Committee (Milan, Italy) approved and authorized the research protocol (37/INT/2018, NCT04076852), in compliance with current national and international laws and regulations governing the use of human subjects in clinical trials (Declaration of Helsinki of 1964). Each participant signed the ethical consent after being informed about all aspects of this study that were crucial for the subject’s decision to participate. The importance of this study for the advancement of medical and psychological knowledge, the aim of this study, the procedures involved, the expected duration of this study, and the need to use sociodemographic data for the purpose of this study were clarified. Furthermore, all participants were reassured about the privacy of their personal data. The informed consent was signed by a parent (or caregiver) of a patient if he/she was a minor.

### 2.3. Statistical Analysis

The collected data were categorized into four seasons of the first year and two seasons of the second year. IBM SPSS Statistics 22 was used for analysis. Since Y-GTSS severity showed a consistent linear decreasing trend across seasons for most patients, individual Y-GTSS scores were detrended. Y-GTSS data normality was assessed by Q-Q plots and the Kolmogorov–Smirnov test. Repeated measures ANOVA (rANOVA) was performed, followed by post hoc analysis for multiple comparisons with Bonferroni correction. Because of the distribution characteristics of Y-BOCS scores (presence of many zeros and skewness), we opted to assume a negative binomial probability distribution and fitted a generalized linear mixed model (GLME) with a logarithmic link function [12]. Multiple mixed linear models (MLEs) were fitted to our data, followed by ANOVA to test the fixed effect of temperature, humidity, age group (minors vs. adults), and semester (hot vs. cold season). Random and repeated factors were included to account for subject variability and repeated measures, respectively. Detrended Y-GTSS scores were used for auto- and cross-correlation analysis, and 7 subjects were excluded for missing data. Spline interpolation was used to facilitate visual interpretation and identify the oscillation period. Cluster analysis was used with two parameters as follows: (1) the lag at which maximum correlation occurred between Y-GTSS scores and local temperature and (2) the maximum value of the correlation observed in the above cross-correlation measure. The following non-parametric tests were adopted: the Mann–Whitney U test for independent samples (first cluster analysis) and the Kruskal–Wallis test for independent samples (second cluster analysis).

## 3. Results

### 3.1. Progression of Tic and OCD Symptomatology along Seasons in Patients with Obsessive–Compulsive Tic Disorder

Table 1 shows the descriptive statistics for the sociodemographic data and for the measurements collected in each season using the three scales (Y-GTSS, Y-BOCS, and Social Impairment Y-GTSS) for the cohort of patients involved in this study (Table 1).

**Figure 2:**
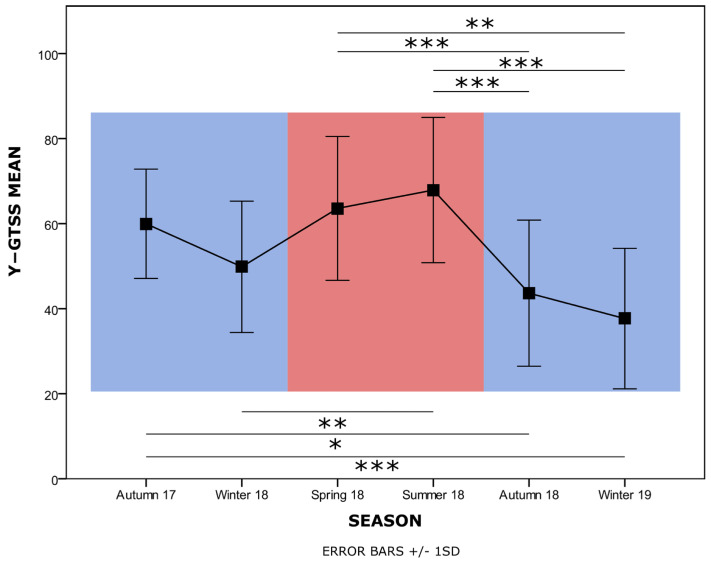
Y-GTSS scores along seasons. Mean values and standard deviation for Y-GTSS measurements over a period of 15 months with quarterly sampling are shown. Background colors represent colder (blue) vs. warmer (red) semesters. Horizontal bars above and below the graph represent significant differences among seasons according to post hoc multiple comparisons (Bonferroni correction). * *p* < 0.05, ** *p* < 0.01, *** *p* < 0.001.

The mean and standard deviation of the Y-GTSS scores obtained along the different seasons from all the subjects of this study are shown in Figure 2.

rANOVA revealed a significant effect of season on Y-GTSS scores (F_(5,19)_ = 11.449, *p* < 0.001; Figure 2). The results of multiple comparisons among seasons (Bonferroni correction) are shown in Figure 2, and the *p*-values are summarized in Table 2.

**Figure 3:**
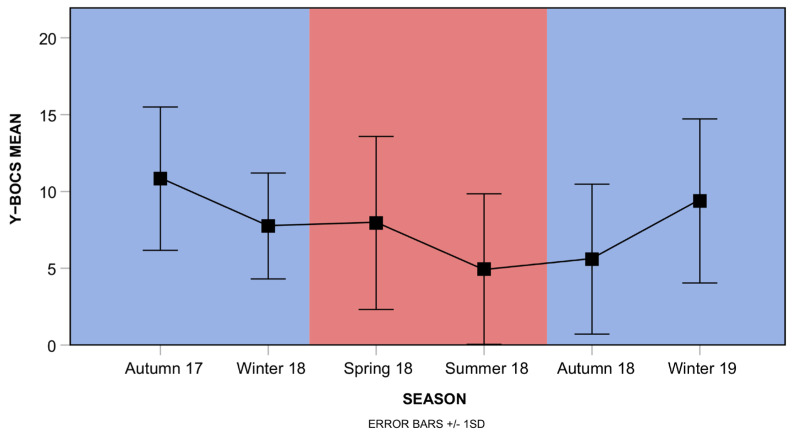
Y-BOCS scores along seasons. Mean values and standard deviation for Y-BOCS measurements over a period of 15 months with quarterly sampling are shown (main effect of season: F_(4,122)_ = 33.490, *p* < 0.001, ANOVA on GLME; all pairwise contrasts with Bonferroni correction n.s.). Background colors represent colder (blue) vs. warmer (red) semesters.

From this post hoc analysis, it emerges that a higher level of symptoms is detected in the warmer seasons (spring–summer), together with an overall decreasing trend, suggestive of an amelioration. Regarding Y-BOCS data, a trend can be appreciated across different measurements (Figure 3), and the ANOVA analysis performed on the GLME detected a significant effect of season, although pairwise comparisons resulted significant (F_(4,122)_ = 33.490, *p* < 0.001; all pairwise contrasts with Bonferroni correction n.s.; Figure 3).

In addition, neither age group nor warm semester met the significance threshold. Therefore, our analysis did not detect an oscillatory dynamic in OCD symptoms.

### 3.2. Effects of Environmental Variables on Seasonal Changes in Tic and OCD Symptomatology

Y-GTSS scores strongly correlated with temperature (r = 0.525, *p* < 0.001) and weakly correlated with Y-BOCS scores (r = 0.175, *p* = 0.047). No significant correlation was found between Y-GTSS and humidity. Y-GTSS scores negatively correlated with the age of subjects (r = −0.198; *p* = 0.025).

The individual range of corrected Y-GTSS was not significantly correlated with age, the Y-GTSS first score (Autumn 2017), or Y-BOCS average scores. Using a linear mixed model (LME) analysis, we found the effect of temperature to be significant (F_(1,14.147)_ = 24.285, *p* < 0.001). Keeping the same random and repeated effects, a second model was developed that included humidity as a fixed effect, which showed no significance for humidity. Since we found both a strong correlation between Y-GTSS and temperature and a milder correlation between temperature and humidity (r = −0.378, *p* < 0.001), we speculate that humidity covaries with temperature but does play a major role in TS symptomatology. As a confirmation, we fitted a third LME including temperature, humidity, and their interaction as fixed effects, which did not detect any significance for the evaluated fixed effects. Lastly, we assessed whether the age group and the semester of the year can account for differences among Y-GTSS scores. We fitted another LME using a dummy variable “hot season” for grouping warmer seasons and the age group (adults vs. minors), with subject as a random effect and season (nested in hot season) as a repeated effect. While season (F_(4,15.239)_ = 19.081, *p* < 0.001) and hot season (F_(1,32.022)_ = 55.874, *p* < 0.001) both resulted significant, confirming the previous results, age group did not, suggesting that age does not exert a significant effect on tic severity.

### 3.3. Seasonal Periodicity in Y-GTSS Scores as Assessed by Autocorrelation and Cross-Correlation Analysis

**Figure 4:**
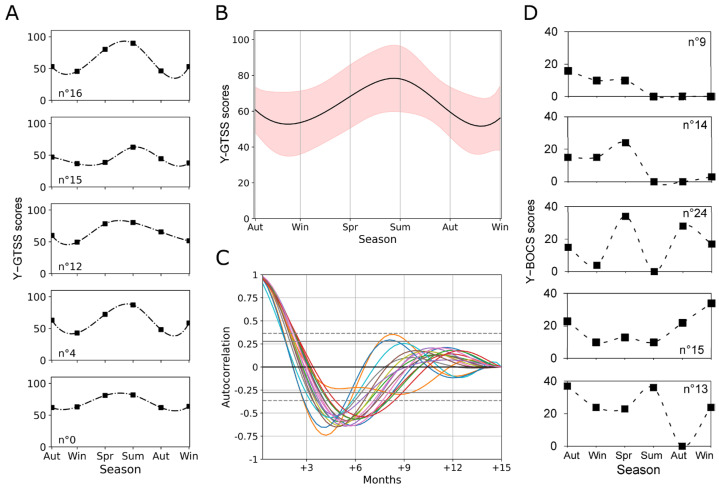
Autocorrelation analysis of seasonal Y-GTSS scores. (**A**) Five individual corrected series of Y-GTSS measurements are shown across the six seasons. The dashed line represents a spline interpolation (50 time points) of the 6 data points. (**B**) Mean (black line) and standard deviation (red area) of corrected and interpolated Y-GTSS data from the 17 subjects selected for autocorrelation analysis. (**C**) Autocorrelation of the individual series of Y-GTSS scores for each patient (random colors). Dashed and straight grey lines indicate 95% and 99% confidence intervals, respectively. (**D**) Five individual trends in Y-BOCS scores across our measuring time span. In comparison with Y-GTSS individual trends in (**A**), Y-BOCS scores do not show a clear oscillatory pattern across seasons.

Figure 4A shows an exemplary corrected series of Y-GTSS scores along the seasons with the 50-point interpolation, while Figure 4B shows a synchronized average (with standard deviation as a red area around the mean) of all series pooled from the 17 subjects selected for autocorrelation and cross-correlation analysis (see Section 2).

The autocorrelation function at successive lags was computed for each corrected series of Y-GTSS scores. As can be appreciated by looking at the correlogram in Figure 4C, for most patients (59%), a negative peak in the autocorrelation function (|r| ≥ 0.6) was found at lag 2 (equal to 6 months). This result indicates that the current Y-GTSS scores are close to those measured after two seasons, which supports the presence of a cyclical trend with a period of about six months. Just 12% of the patients showed high autocorrelation (|r| ≥ 0.5) at lag 1 (three months), indicating that the current Y-GTSS scores are close to those measured in the previous season. Most envelopes also exhibit a rapid decrease in correlation at higher lags. In contrast, individual trends in Y-BOCS scores do not show a clear alternation pattern across seasons (Figure 4D). For this reason, we restricted the synchronization analyses only to tic symptomatology.

**Figure 5:**
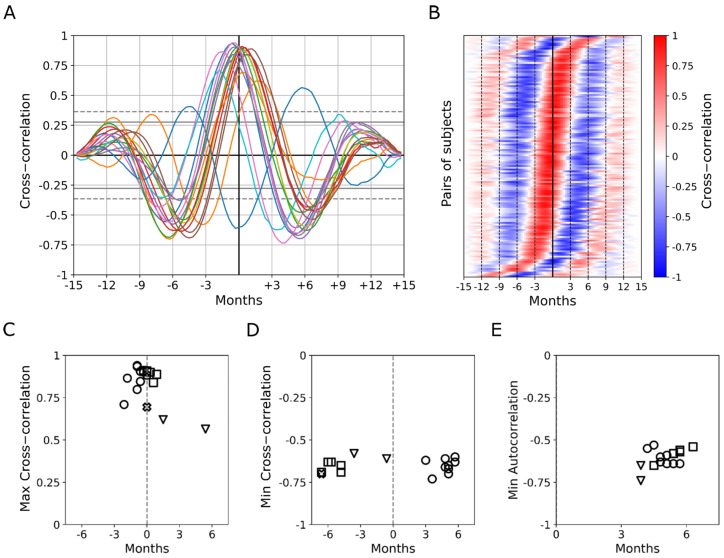
Cross-correlation analysis for detrended seasonal Y-GTSS series. (**A**) Cross-correlation between the series of Y-GTSS scores (50 points interpolation) and the associated temperature series for each subject (random colors), where the x-axis represents lag and the y-axis represents the level of correlation. Dashed and straight grey lines represent 95% and 99% confidence intervals, respectively. (**B**) Cross-correlation between each subject’s detrended series of Y-GTSS scores (50-point interpolation). The color grade represents the level of correlation (blue and red are negative and positive values, respectively). The vertical sorting (from top to bottom) was based on the lag of maximum correlation (from the largest to the smallest lag). (**C**) Maximum values of the cross-correlation between the Y-GTSS score series and temperature series are shown as a function of their lag (in months). (**D**) Negative peaks of the cross-correlation between the temperature series and Y-GTSS series are shown as a function of their lag (in months). (**E**) Negative peaks of autocorrelation in the Y-GTSS series are shown as a function of their lag (in months). Different marker shapes for plots C-E indicate three different clusters obtained using cluster analysis.

The cross-correlation function was then computed between the series of Y-GTSS scores from each couple of different patients (corrected and interpolated series as for the autocorrelation analysis). The functions are shown in Figure 5A (random colors).

As it can be appreciated, the peaks of these functions (|r| ≥ 0.6) in most cases occur at lags very close to 0, indicating that the oscillations in symptoms along different seasons are highly synchronized for most patient couples. Interestingly, several couples of patients also show negative peaks in the cross-correlation function (r ≤ −0.6) at lag ±2 (equal to 6 months).

In some instances, the maximum in correlation (|r| ≥ 0.6) can be found at lags close to ±1, which indicates a rare shift in the seasonal oscillation period; the cross-correlation functions were sorted as a function of the peak’s lag in Figure 5B, to better show such “phase” variability. Cross-correlation between the Y-GTSS score series and temperature series over seasons produce positive peaks at lag 0 and negative peaks at lag 2 for 65% of the patients, indicating a relationship between the seasonal oscillations in Y-GTSS symptoms and those in ambient temperature. Nevertheless, from this analysis, it emerges that 12% of the patients show a strong positive correlation at lag 1 and a strong negative correlation at lag 3, indicating that the Y-GTSS oscillation might anticipate the temperature oscillation by one season. The remaining 18% of the patients have an irregular correlation pattern over lags. Finally, the cross-correlation analysis between the Y-GTSS and Y-BOCS scores series did not allow us to identify a unique correlation pattern since the results were highly variable.

### 3.4. Different Patterns in Seasonal Periodicity Emerge in Tic and OCD Symptomatology

To evaluate if the oscillatory behavior arose mainly in limited subgroups of patients, we conducted two cluster analyses on the Y-GTSS data. The first cluster analysis for the classification of patients (see Section 2) suggests the presence of two clusters based on a measure of synchronicity between Y-GTSS and temperature seasonal oscillations. Cluster 1 had a total of 14 subjects and was defined by high synchronicity, whereas cluster 2 had just 3 subjects characterized by low synchronicity between Y-GTSS and temperature. There was no significant difference between clusters with respect to Y-GTSS at t = 0, Y-GTSS range, Y-BOCS mean, age, or temperature deltas over seasons (all *p*-values above the threshold, Mann–Whitney U test). As for Y-GTSS deltas over seasons, a statistically significant difference was found only in summer–autumn and in autumn–winter Y-GTSS deltas (*p* = 0.047 and *p* = 0.032, respectively, Mann–Whitney U test).

The cluster analysis regarding the second classification of patients suggests four clusters on a measure of Y-GTSS delta values between subsequent seasons. Cluster 1 comprised nine subjects, cluster 2 comprised only one, cluster 3 included six subjects, and cluster 4 had only one subject, sharply distant from the other three clusters. There was no significant difference among these clusters with respect to Y-GTSS at t = 0, Y-GTSS range, Y-BOCS mean, age, or temperature deltas over seasons (all *p*-values above the threshold, Kruskal–Wallis test).

## 4. Discussion

As with the great majority of neuropsychiatric disorders, the mechanisms underlying Tourette syndrome are still largely unknown. The brain circuits involved are presumably those located in the basal ganglia since anatomical evaluations have found volume loss and white matter changes in these nuclei [13,14]. A working hypothesis for this condition indicates a disinhibition of cortico-basal ganglia loops (motor, sensory, and limbic) as the pathogenetic mechanism, also supported by the efficacy of brain stimulation in ameliorating tics expression [15]. This might derive from a change in the dopaminergic pathways as suggested by the efficacy of pharmacological agents used [16].

Our study provides clear evidence for the role of environmental temperature on tic severity in patients with TS. This result is supported by both seasonal effects on tics, which include tic exacerbation with warmer seasons, and a significant correlation between tic symptomatology and external temperature, independently from humidity and patient age. In addition, our analysis detected cyclic behavior in tic severity, with a period of about six months and high inter-subject synchronicity. In most cases, such cyclic dynamics seem phase-locked to temperature fluctuations. These results are in line with those from past studies that investigated the effects of external temperature challenges on tic frequency in TS patients [17,18]. In those reports, temperature was found to promote tics. In particular, the study by Scahill et al. (2001), which involved a large cohort of TS patients, found that almost one-fourth of the patients increased tic frequency following a rise in external temperature of 13 °C [18]. Furthermore, deficits in body temperature regulation have been detected in TS patients [19]. Interestingly, the existence of a relationship between environmental temperature and the exacerbation of symptoms can be generalized, as it has been observed for several disorders. Bundo and colleagues (2021) found a 4% increase in hospitalizations for each 10 °C increase in environmental temperature, independently of gender. Interestingly, such an effect seems stronger for psychiatric and developmental disorders [20]. The cyclic behavior we observed cannot be ascribed to group A streptococcus infections because the patients were monitored for any infections and, if detected, promptly treated with antibiotic therapies.

In our settings, although a significant temporal trend could be appreciated, no significant difference between warmer and hotter seasons could be detected from our analysis of obsessive–compulsive symptoms assessed by the acquisition of Y-BOCS scores. This result, together with the evaluation of individual temporal courses in Y-BOCS scores, suggests that in our cohort of patients, a significant seasonal oscillatory pattern in OCD symptomatology can be excluded. It is worth noting that in a previous study from our group, a seasonal variation in obsessive–compulsive symptoms was detected, albeit by using a different analysis approach [9]. An explanation of such ambiguity might come from the limitations in Y-BOCS data collection: on the one hand, such symptoms are much more difficult to assess and quantify based on subjective observation, compared with motor and vocal tics (that can be counted as discrete episodes), and this is especially true when scoring is performed by parents of pediatric patients. On the other hand, it is important to highlight that a floor effect can be appreciated in our data (several zeros), indicating that the severity of OCD symptoms in our cohort was very low, or at least under-rated, thus not providing enough sample variability to detect seasonal oscillations.

Based on our evidence of clear temperature-related seasonal oscillations in tics in our cohort of TS patients, the role of the hypothalamus immediately comes to mind since its nuclei receive thermal sensory information from the periphery, collected by specific subtypes of transient receptor potential ion channels (TRPs). The hypothalamus regulates all autonomic (e.g., vasomotor and sudomotor) and behavioral motor responses [21] that contribute to regulating body temperature in different environmental conditions. More specifically, the preoptic ventral as well as dorsomedial region of the hypothalamus seem to be involved in such sensory-driven maintenance of body temperature homeostasis inside a tiny range (~36–39 °C) [22]. Related to this, Kessler (2002) detected a strong misperception of ambient temperature by TS patients and hypothesized a hypothalamic disorder [19]. In this context, an important aspect relates to the effects of dopamine and norepinephrine signaling on hypothalamic circuitry, with a major role of D2 receptors [22], as Tourette etiopathology and tic expression are thought to involve dopaminergic system dysregulation in the basal ganglia. Indeed, pharmacological treatments of TS mostly involve dopaminergic antagonists that were shown to affect thermoregulation and metabolism dramatically, and other neurological conditions affecting dopamine transmission, such as Parkinson’s disease, present thermoregulation anomalies as well [23]. Nevertheless, the effects of antipsychotic medications known to have dopaminergic modulatory action can be rather complex, involving both hypothermia and hyperthermia depending on specific conditions [22]. Accordingly, studies involving different animal models lead to contradictory results about the effects of dopamine on body temperature regulation. In the rat, dopaminergic agonists either reduce or increase body temperature based on external temperature conditions [24], while this treatment induces only hyperthermia in the rabbit, with a more complex effect of D2 antagonists [25].

The external temperature might also affect human motor behavior through systemic changes in dopaminergic modulation. Such a view is in line with the physiological mechanisms of motor behaviors aimed at body temperature regulation [21], which rely on the complex and intermingled circuitry connecting the hypothalamus to dopaminergic nuclei, including both ventral tegmental area (VTA) and substantia nigra (SN), through the channeling action of the rostromedial tegmental nucleus (RMTg; also known as the tail of the VTA). Interestingly, dopamine levels were recently found to be enhanced by artificial increases in environmental temperature in humans [26], while seasonal changes were shown to exert significant effects on dopamine synthesis in the human brain, with special reference to the striatum, but in the opposite trend [27]. Such changes in dopamine might either act directly on the mechanisms supporting tic expression, or indirectly, by modulating the activity and plasticity of neural circuits at several locations [28,29]. Another possible explanation for the thermoregulation deficits and susceptibility of tics to the environmental temperature might relate to some impairments in hypothalamic functions, as previously described [19]. Nevertheless, such a view still lacks a reasonable link to Tourette etiopathology. A possible explanation might lie in the specific anatomical features of the hypothalamus. Future investigations could investigate other environmental variables such as daylight luminance, which influences visual sensory processing; indeed, sensory gating is thought to be altered in TS, supported by thalamic morphological anomalies and the complex filtering abilities of thalamic circuits [30,31].

Interestingly, the ventrobasal hypothalamus is known to be easily reached by blood substances because of the lack of a blood–brain barrier (BBB) in its median eminence [32]. Such an aspect renders the hypothalamic nuclei particularly vulnerable to the action of circulating molecules. Auto-antibodies developed following streptococcus group A streptococcus infections are supposed to contribute to TS development [33], in analogy with other pediatric autoimmune disorders associated with streptococcal infections (PANDAS), as demonstrated by the presence of anti-basal ganglia antibodies [34,35]. Therefore, these auto-antibodies and other neuromodulatory molecules might act upon the hypothalamus and disturb its ability to sense and regulate temperature. This might lead to the enhanced sensitivity of tics to environmental temperature changes and therefore to the seasonal oscillations here found.

### Limitations and Future Developments

An observational longitudinal study was conducted on a cohort of 24 people recruited in Milan (Italy) and diagnosed with a subtype of TS known as obsessive–compulsive tic disorder. The selection of this subtype partially limited the recruitment process, but a more impacting factor was the large drop-out, likely due to the long timeframe of this study and the large number of sequential evaluations. Future studies should try to overcome this issue in order to verify whether the oscillation pattern can be generalized to the clinical population and also to a longer timeframe.

Further studies are also required to evaluate all the explicatory hypotheses previously mentioned. On a clinical level, our study indicates that seasonal environmental changes should be considered for patients’ treatments as well as when evaluating their clinical course. This could greatly improve the efficacy of pharmacological treatments, which in turn can minimize dosages, thus reducing the impact of side effects.

## 5. Conclusions

Our results indicate that the increase in tics observed during hot seasons can be related to increasing ambient temperature. We believe that our results can shed light on the seasonal dynamics of TS symptomatology and provide clues for preventing their worsening over the year, as well as novel insights for understanding the physiopathology of this still obscure and invalidating syndrome.

## Figures and Tables

**Table 1:** Sociodemographic and seasonal data for the Y-GTSS and Y-BOCS scales for the cohort of patients involved in this study. Y-GTSS: Yale global tic severity scale; Y-BOCS: Yale–Brown obsession and compulsion scale; social impairment represents a subscale of the Y-GTSS. Scale results are presented as mean ± SD.

Sociodemographic Data			
**N**	24		
**Sex (male–female)**	20:4		
**Age (mean ± SD)**	20.58 ± 9.18		
	**Y-GTSS**	**Y-BOCS**	**Social Impairment Y-GTSS**
**Autumn 2017**	59.96 ± 12.85	10.75 ± 11.04	33.33 ± 7.61
**Winter 2018**	49.83 ± 15.44	8.21 ± 8.60	28.54 ± 8.90
**Spring 2018**	63.58 ± 16.92	8.53 ± 12.06	33.25 ± 12.95
**Summer 2018**	67.89 ± 17.08	6.71 ± 11.41	36.40 ± 11.78
**Autumn 2018**	43.64 ± 17.19	6.15 ± 11.41	2.11 ± 13.18
**Winter 2019**	37.67 ± 16.52	8.95 ± 11.62	18.14 ± 13.40

**Table 2:** Post hoc analysis for repeated measures analysis on Y-GTSS data. Only significant *p*-values (*p* < 0.05) are reported for each comparison (Bonferroni correction). Background colors represent colder (blue) vs. warmer (red) semesters.

Significant *p*-Values	Autumn 17	Winter 18	Spring 18	Summer 18	Autumn 18	Winter 19
Autumn 17					0.011	<0.001
Winter 18				0.005		
Spring 18					0.002	<0.001
Summer 18		0.005			<0.001	<0.001
Autumn 18	0.011		0.002	<0.001		
Winter 19	<0.001		<0.001	<0.001		

## Data Availability

The data supporting the findings of this study are available from the corresponding author upon reasonable request.
